# Individuality decoded by running patterns: Movement characteristics that determine the uniqueness of human running

**DOI:** 10.1371/journal.pone.0249657

**Published:** 2021-04-01

**Authors:** Fabian Hoitz, Vinzenz von Tscharner, Jennifer Baltich, Benno M. Nigg

**Affiliations:** 1 Biomedical Engineering, Schulich School of Engineering, University of Calgary, Calgary, Alberta, Canada; 2 Human Performance Laboratory, Faculty of Kinesiology, University of Calgary, Calgary, Alberta, Canada; 3 Brooks Sports Inc., Seattle, Washington, United States of America; University of Illinois at Urbana-Champaign, UNITED STATES

## Abstract

Human gait is as unique to an individual as is their fingerprint. It remains unknown, however, what gait characteristics differentiate well between individuals that could define the uniqueness of human gait. The purpose of this work was to determine the gait characteristics that were most relevant for a neural network to identify individuals based on their running patterns. An artificial neural network was trained to recognize kinetic and kinematic movement trajectories of overground running from 50 healthy novice runners (males and females). Using layer-wise relevance propagation, the contribution of each variable to the classification result of the neural network was determined. It was found that gait characteristics of the coronal and transverse plane as well as medio-lateral ground reaction forces provided more information for subject identification than gait characteristics of the sagittal plane and ground reaction forces in vertical or anterior-posterior direction. Additionally, gait characteristics during the early stance were more relevant for gait recognition than those of the mid and late stance phase. It was concluded that the uniqueness of human gait is predominantly encoded in movements of the coronal and transverse plane during early stance.

## Introduction

The ability to identify individuals based on their movement patterns (gait recognition) has long fascinated society [1]. In biomechanics and biometrics, the mechanisms of human gait have been explored as biometric features and it has been demonstrated repeatedly that human gait is as unique to an individual as is their fingerprint [2–4]. Pataky and colleagues [5], for instance, showed that 104 individuals could be differentiated based only on their dynamic plantar pressure patterns with accuracy rates of over 99%. Gait patterns derived from joint angle trajectories and ground reaction forces (GRF) have also been explored to distinguish between individuals and resulted in comparable accuracy rates [6].

For many of these findings, the methodological approach was centered around machine learning procedures such as support vector machines [7, 8] and neural networks [9, 10]. A major benefit of these techniques is that they can handle the large datasets that are required for the non-trivial task of gait recognition. In the aforementioned work by Pataky and colleagues [5], the data for one participant included 94,250 data points. Due to these large data volumes, however, machine learning models become very complex and require an extensive (and time consuming) computational effort to extract valuable information [11]. Additionally, these models act as a black box, providing no indication as to what information they deemed relevant to their decision-making [12, 13]. Thus, there remains a lack of knowledge on what part of the original data may be relevant for a successful classification by the model. In biomechanics this lack of transparency is a major drawback as it is often more important to understand *when* and *why* datasets differ rather than *whether* they are different at all. Despite the consensus that human gait is unique, it remains unknown *what* gait characteristics differentiate well between individuals and determine the uniqueness of human gait patterns.

Efforts have been made to develop methodologies that unveil a model’s decision-making process [14]. One method in particular, layer-wise relevance propagation [15], has successfully been applied in the contexts of image classification [16], text-document classification [17], and recently in gait classification [6]. In brief, layer-wise relevance propagation (LRP) assigns a relevance to each variable that indicates its contribution to the classification result. Combining layer-wise relevance propagation with machine learning, therefore, promises to increase the transparency of complex models. For a detailed explanation on layer-wise relevance propagation, the reader is referred to the original work by Bach and associates [15]. In the context of gait recognition, layer-wise relevance propagation quantifies the extent to which a specific gait characteristic contributes to the recognition of an individual. It may further provide the means to reduce the required data, which may facilitate a functional interpretation of gait characteristics that may be relevant for gait recognition.

The aim of this work was to explore what gait characteristics and time points of stance differentiate well between individuals and determine the uniqueness of human gait. Specifically, the purposes of this work were:

1. To train a neural network model to distinguish individuals based on their movement patterns observed by high-speed video of reflective markers and measured ground reaction forces after an eight-week intervention period.
2. To identify those variables and time points of overground running movement patterns that are most relevant to the identification of individuals using layer-wise relevance propagation.
3. To explore layer-wise relevance propagation as a means for data reduction and compare the performances of neural networks trained on only those variables with high relevance.

## Methods

The present work resulted from a secondary analysis of previously collected data. For a detailed description of the protocol and the primary purpose of the data, the reader is referred to the original work [18].

### Participants

Fifty participants (12 males [mean ± std]: 33.5 ± 8.9 years, 95.6 ± 27.2 kg, 179 ± 6.4 cm; 38 females [mean ± std]: 35.2 ± 9.4 years, 69.8 ± 13.2 kg, 164.7 ± 6.3 cm) were recruited for this study. All participants were novice recreational runners with less than 2 years of running experience and no symptoms of pain or injury within three months of testing. Written informed consent was collected from all participants and approval for this research project was obtained from the ethics board of the University of Calgary (ethics ID: REB 13–0153).

### Data collection

Participants performed 20 overground running trails at 3.5 m/s (± 15%) along a 30 m long indoor runway on two days, eight weeks apart. For the eight-week period between testing days, participants were randomly assigned to one of three intervention groups: control, functional balance training, or strength training. All three intervention groups were required to perform a home-based exercise routine five times per week. The exercise routine consisted of aerobic activities, statics stretching, and dynamic stretching. Members of the control group performed said exercise routine for 25 minutes each session, while members of the strength and balance groups performed this routine for 5 minutes followed by a 20-minute specialized exercise protocol for their respective group. For the detailed exercise protocols the reader is referred to the original work by Baltich and colleagues [18].

On both testing days, kinematic data were collected using an eight high-speed video camera system (Motion Analysis Corporation, Santa Rosa, Ca, USA) at a sampling rate of 240 Hz. Clusters composed of three reflective skin markers (diameter: 12.7 mm) were placed on each of the three segments of the right lower extremity (thigh, shank, and foot) and an additional four markers were placed on the right and left anterior / posterior iliac spine. Kinetic data was collected simultaneously using a force plate (Kistler Instruments AG, Winterthur, Switzerland) embedded within the laboratory floor, sampling at a frequency of 2400 Hz. Prior to the overground running trials, a standing trial was collected to obtain baseline measurements for marker positions, and the appropriate starting position for each participant was determined so that a single step on the force plate was guaranteed. The axes of the global coordinate system for the motion capture system were defined in such a way that the x-axis pointed in the direction of running, the y-axis was defined as perpendicular to the running direction, and the z-axis was directed vertically upwards.

### Analysis

The number of overground running trials that were analyzed varied across participants because steps that almost missed the force plate were excluded. A minimum of 29 running trails, however, was guaranteed, while a maximum of 40 (20 per day) was possible. Trajectories of 3D marker positions were reconstructed using Expert Vision 3D Analysis software (Motion analysis Corporation, Santa Rosa, CA, USA), and cut to the stance phase of the stride that occurred on the force plate. The stance phase was defined as the period between heel strike and toe off, and both events were identified using a 15 N threshold applied to the vertical GRF. For each sample point, the rotation relative to the standing position for the hip, thigh, shank, and foot segment was calculated based on the marker clusters located on the respective segments [19]. Subsequently, 3D joint angles for the hip, knee, and ankle were calculated as the relative rotation between the hip and thigh, the thigh and shank, and the shank and foot segment, respectively, using a Z-X-Y cardan rotation sequence. The resulting joint angle trajectories and GRF trajectories were smoothed with a 15 Hz and a 50 Hz wavelet-based lowpass filter [20], respectively. Finally, an extensive normalization procedure was applied to each trajectory: First, all trajectories were time-normalized to 100 values via shape-preserving piecewise cubic interpolation. Second, the average trajectory of the respective participant was subtracted, and the given trajectory was divided by the standard deviation of the respective participant. Last, all trajectories were rescaled to range from -1 to 1 and concatenated into a single feature vector of dimensions 1 x 1200 (12 trajectories x 100 samples), representing the movement pattern of a single stride (stride pattern). Through this procedure effects, such as differences in body weight, were eliminated from the data.

A shallow neural network was designed that consisted of one input, one hidden, and one output layer with 1200, 2400, and 50 nodes, respectively. A single hidden layer is, reportedly, sufficient to learn most input-output relationships [12], and the nodes per layer were derived from the shape of the input data (i.e., 1200 variables per stride pattern and 50 participants). The hyperbolic tangent was used as an activation function for the hidden layer. The network was trained on the entire data set collected during day 1, with a batch size of 25 and an epoch limit of 3000, which were determined to be a viable tradeoff between training efficiency and model accuracy. The data set collected during day 2 was subsequently presented to the trained model to assign the stride patterns of day 2 to their respective individual. The networks performance on the unseen data of day 2 was determined by calculating the participant-wise accuracy using Eq 1, where, for a given participant *p*, *n* described the number of correctly assigned stride patterns and *N* the total number of stride patterns.

$$Accuracy(p)=\frac{n_{p}}{N_{p}}*100$$ (1)

For each stride pattern that was correctly assigned by the neural network model, individual relevance patterns were calculated using layer-wise relevance propagation. By assigning a relevance score to each input variable, layer-wise relevance propagation determined how relevant each variable was to the model’s prediction. The relevance patterns derived by layer-wise relevance propagation were then normalized to their respective maximum to enable comparisons between participants. Subsequently, the average across all relevance patterns was calculated and rectified. The rectified average was smoothed, whereby the previous and subsequent points were weighted with 25%, and the current point with 50%. This smoothing process was repeated three times. Finally, the smoothed relevance pattern was rescaled to range from 0 (lowest relevance) to 1 (highest relevance). Because the input data (i.e., step patterns) were collected in the time-domain, neighboring values were dependent and represented related information. The applied smoothing process, therefore, reduced fluctuations in the calculated relevance scores without affecting the general pattern. The weights for the smoothing process were chosen such that their sum equaled 1 and a repetitive application would mimic a gaussian filter.

To explore the effect of the individual variables on the average classification accuracy of the model, all variables were sorted according to their relevance. A threshold was applied to limit the number of variables that should be included in a reclassification of all stride patterns of day 2. This threshold was increased in an iterative procedure such that an increasing subset of variables was used for the reclassification. Variables excluded from the various subsets were set to 0.

All analysis was performed in MATLAB 2019b (Natick, Massachusetts: The MathWorks Inc.) and the layer-wise relevance propagation toolbox by Lapuschkin and colleagues [21] was used for all analyses based on layer-wise relevance propagation. The supporting information of this work (S1 File) contains all analysis codes (MATLAB) and data files required to replicate the results presented in this work.

## Results

**Fig 1:**
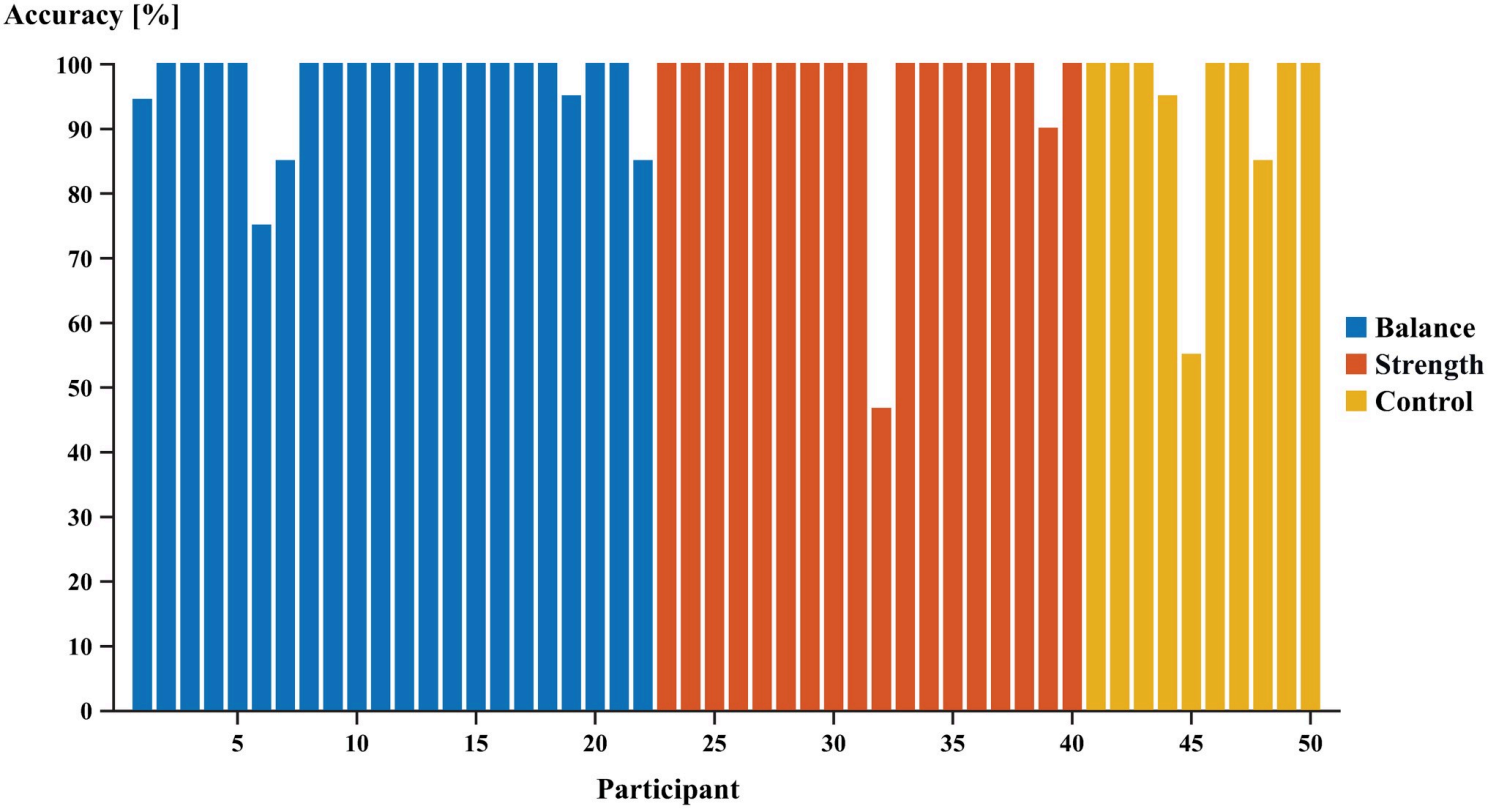
Classification accuracies of the trained neural network on unseen data, stratified by participants and intervention groups.

The model that was trained on the day one stride patterns of all participants correctly assigned the day two stride patterns to the respective participants with an accuracy of 96.1%. For most participants (44 / 50), the model’s accuracy was ≥ 90% (Fig 1). For four participants the accuracy of the model was between 70% and 90%, and for two participants the model’s accuracy was below 60%. The model performed equally well irrespective of the intervention (balance, strength, control) that was performed between day 1 and day 2.

**Fig 2:**
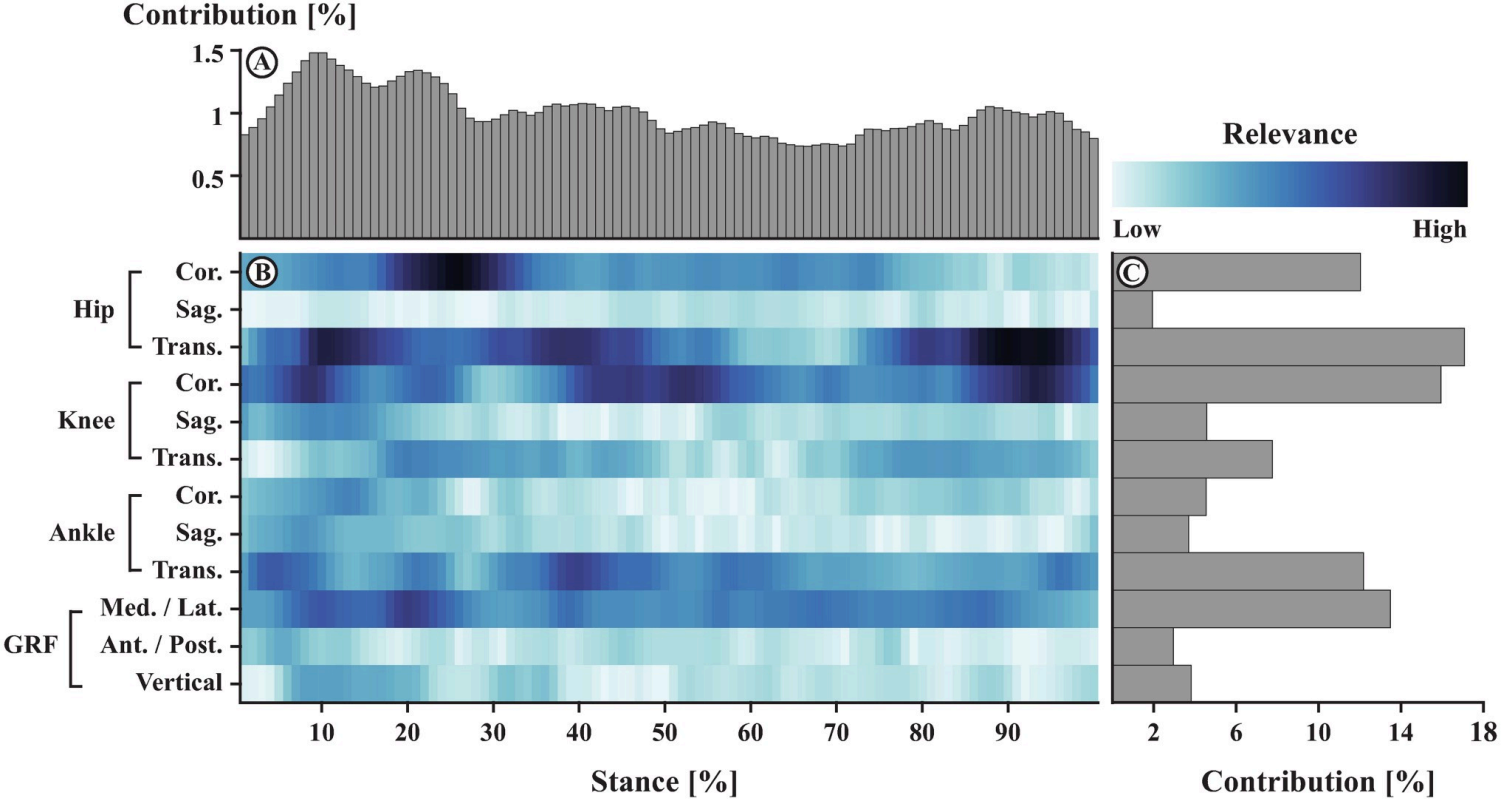
Absolute relevance of each variable within a stride pattern averaged across all relevance patterns. The top part (A) shows the summed contribution of relevance for each of the 100 time points of stance. In the center (B), darker colors indicate variables of high relevance, while lighter colors indicate variables of low relevance. In other words, to assign a stride pattern to the respective participant, the model relied more on variables with darker shades. Variables with lighter shades were less relevant for a correct classification of gait patterns. The right part of the figure (C) highlights the summed contribution of relevance of each direction of joint angle trajectories.

The variables recorded at each 1% interval of stance phase were all relevant to successfully match stride patterns to their respective individuals (Fig 2A). The contribution of variables from the early stance phase (1–30%) to the successful classification, however, was higher than the contribution of variables from the mid and late stance phase.

The variables of trajectories that were most relevant were ab-adduction and internal-external rotation of the hip, the ab-adduction of the knee, the in-eversion of the ankle, and the medio-lateral GRF (Fig 2C). On the contrary, the trajectories that were least relevant to a successful matching of stride patterns and individuals were the flexion-extension of the hip, the flexion-extension and internal-external rotation of the knee, the ab-adduction and dorsi-plantarflexion of the ankle, and the GRF in anterior-posterior and vertical direction.

The detailed distribution of relevance (Fig 2B) revealed variables and time points of overground running movement patterns that were most relevant to the identification of individuals.

Notable percentages of stance that were of high relevance included hip ab-adduction at 20%–30% of stance; hip internal-external rotation at 10%–20%, 35%–45%, and 85%–98% of stance; knee ab-adduction at 9%, 45%–55%, and 90%–95% of stance; ankle in-eversion at 5%, 40%, and 95% of stance; and medio-lateral GRF at 20% of stance (Fig 2B, dark shades). On the contrary, percentages of stance that were of low relevance included hip flexion-extension at 1%–10%, 15%–30%, and 90%–100% of stance; knee flexion-extension at 38%–55%, and 97% of stance; knee internal-external rotation at 1%–5%, and 55%–65% of stance; ankle ab-adduction at 26%, 45%–60%, and 98% of stance; ankle dorsi-plantarflexion at 50%–60%, and 75%–95% of stance; anterior-posterior GRF at 25%–30%, and 70%–100% of stance; and vertical GRF at 1%–5%, 40%–50%, and 95% of stance (Fig 2B, light shades).

**Fig 3:**
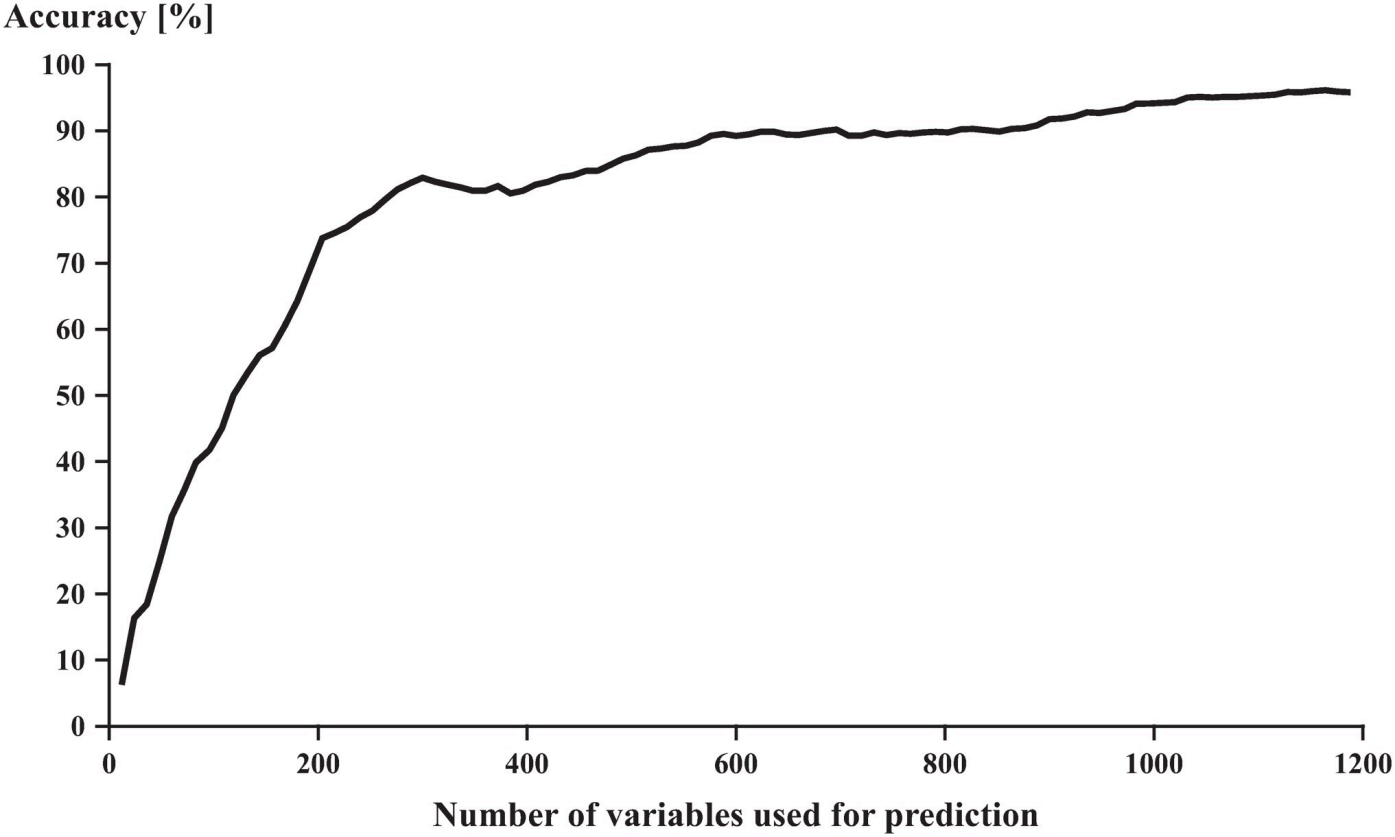
Average classification accuracy as a function of the number of highly relevant variables used for the classification.

The reclassification of day two stride patterns based on subsets of variables with high relevance showed that the relationship between the average accuracy of the model and the number of included variables was non-linear (Fig 3). With an increasing number of highly relevant variables (16–200), the model’s accuracy increased rapidly (5%– 72%). Once the subset of variables included the 200 variables with the highest relevance, however, the model’s accuracy gains from additional variables were smaller. The entire set of variables (1200) was needed to reach the model’s highest accuracy of 96%.

**Fig 4:**
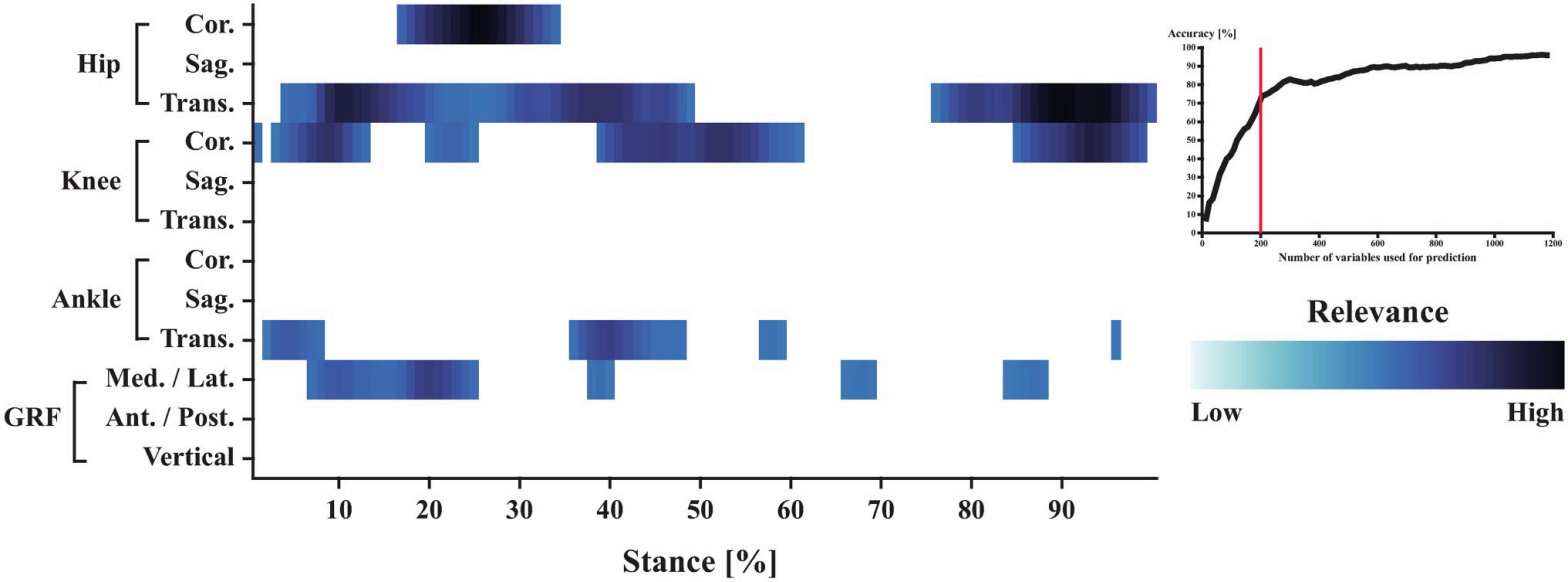
Absolute relevance of the 200 variables with the highest relevance within a stride pattern averaged across all relevance patterns.

Finally, the 200 variables with the highest relevance were exclusive to the trajectories that described the ab-adduction of the hip and knee, the internal-external rotation of the hip, in-eversion, and the medio-lateral ground reaction forces (Fig 4).

## Discussion

The purpose of this work was threefold: First, to explore if a neural network can be trained to recognize individuals based on their gait pattern (i.e., individual strides) after an eight-week intervention period. Second, to use layer-wise relevance propagation to determine those variables of a gait pattern that were more relevant to the classification and those that were less relevant. Third, to explore the model’s performance on subsets of variables of high relevance.

The results of this work showed that a neural network could identify individuals based on their gait patterns (joint angle trajectories and ground reaction forces) with high accuracies (> 90%). This outcome confirms previous findings that reported high success rates when using machine learning techniques (e.g., neural networks, support vector machines, etc.) to identify individuals, and / or functional groups (e.g., young vs. elderly) based on their gait patterns [6, 22–26]. These findings provide support for the paradigm of the preferred movement path [27, 28] which suggests that individuals have a unique and preferred movement pattern. In addition, the present work may suggest that a preferred movement pattern is stable over an eight-week intervention period as the applied functional balance, strength training or stretching did not alter movement patterns to the point where the neural network was unable to match them to the correct individual. The long term stability of movement patterns, as was hypothesized previously [22, 29], might also explain why earlier investigations (using the same data) did not find any effects on injury risks [30–32]. Thus, it appears that an individual’s preferred movement pattern is unique and does not easily change.

From the publications cited above, one can see the overwhelming amount of data that can be recorded and used to identify gait patterns. A reduction of the number of variables to those that are relevant for pattern recognition is essential if one has limited resources. The main contribution of the present work, therefore, lies within the application of layer-wise relevance propagation to the trained neural network model. Layer-wise relevance propagation highlighted the relevance of each variable for a successful assignment of gait patterns to individuals. From Fig 2 it is evident that not all variables (i.e., gait characteristics) were equally relevant. The model ‘learned’ from the training data that some gait characteristics were more discriminative than others. Movements in the coronal and transverse plane, for example, were found to be more relevant for a discrimination of individuals than movements in the sagittal plane (Fig 2C). Similarly, medio-lateral GRFs were more relevant than forces in the anterior-posterior and vertical direction. Further, early stance (i.e., 1%–30%) was more relevant to the success of the model than mid and late stance (Fig 2A).

Intuitively, the higher relevance of some gait characteristics over others may suggest that the uniqueness of a gait pattern is not equally distributed across all gait characteristics. In other words, gait characteristics with low relevance might be generic and thus similar across multiple individuals and, therefore, they were not relevant for gait recognition. Gait characteristics with high relevance, however, might be specific (i.e., unique) to an individual and thus highly relevant for gait recognition. Consequently, one would expect gait characteristics with high relevance scores to differ substantially across individuals and future studies should focus on these.

Previous publications lend support to the outlined concept: For example, Horst and Colleagues (who also utilized layer-wise relevance propagation) showed that the most prominent individual gait characteristics of barefoot overground walking where often located within the early stages of stance [6]. Likewise, it has been shown that differences in foot strike patterns (i.e., rearfoot vs. forefoot strike) can be readily observed during the early stages of stance [33]. Because the current work did not control for strike patterns, it is likely that strike patterns varied across participants and, consequently, the early stance phase might have been more important to discriminate between individuals. Similarly, medio-lateral GRF have been shown to be more variable across individuals than anterior-posterior and vertical GRF [34] and might, therefore, capture more of an individual’s unique gait characteristics or preferred movement pattern. In fact, the present work highlighted that a subset of appropriately selected gait characteristics (i.e., variables with highest relevance) resulted in accuracies close to those when the entire data set was used. Specifically, 200 of the total 1200 gait characteristics proved to be sufficient to achieve a classification accuracy of over 70%, while the entire dataset resulted in an accuracy of 96%. Interestingly, the 200 variables with the highest relevance were specific time points of ground contact within only five of the twelve trajectories (hip ab-adduction and internal-external rotation, knee ab-adduction, in-eversion of the foot, and medio-lateral ground reaction forces; Fig 4). These five trajectories may, therefore, encode the majority of an individual’s unique gait pattern. In fact, when performing gait recognition on only those five trajectories over the entirety of ground contact (i.e., 500 variables), the model’s performance resulted in an accuracy of 75.33%. Future studies concerned with the problem of gait recognition are, thus, advised to focused on the trajectories of hip and knee ab-adduction, hip internal-external rotation, foot in-eversion, and the medio-lateral ground reaction forces as they may provide the majority of information required for successful gait recognition.

This work showcases a framework (layer-wise relevance propagation) for the analysis and interpretation of machine learning models and may provide guidance for future studies on the uniqueness of human gait. However, the relevance scores were a function of the trained model based on the current dataset using novice runners of different sex. It is unclear if similar findings would have been obtained using a different dataset. For example, variables identified as highly relevant may vary across running populations of varying running experience. Future studies that investigate a broader population are, therefore, needed to confirm the outcomes of this work.

## Conclusion

Gait characteristics of the coronal and transverse plane as well as medio-lateral ground reaction forces provided more information on an individual’s unique movement pattern than gait characteristics of the sagittal plane and ground reaction forces in vertical or anterior-posterior direction. From a temporal perspective, gait characteristics during the early stance were more unique than those of the mid / late stance. Thus, the uniqueness of human gait is predominantly encoded in movements of the coronal and transverse plane during early stance.

## Supporting information

S1 File Data and analysis codes. This archive contains all the underlying data (raw and processed) presented in this publication and the respective analysis codes (Matlab) to follow the methodological steps of this work.
(ZIP)

## References

[1] Nixon MS, Tan T, Chellappa R. Human identification based on gait. Boston, MA: Springer US; 2006. 10.1007/978-0-387-29488-9

[2] Dehzangi O, Taherisadr M, ChangalVala R. IMU-Based Gait Recognition Using Convolutional Neural Networks and Multi-Sensor Fusion. Sensors (Basel). 2017;17. 10.3390/s17122735 29186887 PMC5750784

[3] Lin YC, Yang BS, Lin YT, Yang YT. Human recognition based on kinematics and kinetics of gait. Journal of medical and biological engineering. 2011;31: 255.

[4] Weich C, Vieten MM. The gaitprint: identifying individuals by their running style. Sensors (Basel). 2020;20. 10.3390/s20143810 32650424 PMC7412195

[5] Pataky TC, Mu T, Bosch K, Rosenbaum D, Goulermas JY. Gait recognition: highly unique dynamic plantar pressure patterns among 104 individuals. J R Soc Interface. 2012;9: 790–800. 10.1098/rsif.2011.0430 21900318 PMC3284135

[6] Horst F, Lapuschkin S, Samek W, Müller K-R, Schöllhorn WI. Explaining the unique nature of individual gait patterns with deep learning. Sci Rep. 2019;9: 2391. 10.1038/s41598-019-38748-8 30787319 PMC6382912

[7] Yoo J-H, Hwang D, Nixon MS. Gender classification in human gait using support vector machine. In: Blanc-Talon J, Philips W, Popescu D, Scheunders P, editors. Advanced concepts for intelligent vision systems. Berlin, Heidelberg: Springer Berlin Heidelberg; 2005. pp. 138–145. 10.1007/11558484_18

[8] Xue Z, Ming D, Song W, Wan B, Jin S. Infrared gait recognition based on wavelet transform and support vector machine. Pattern Recognit. 2010;43: 2904–2910. 10.1016/j.patcog.2010.03.011

[9] Shiraga K, Makihara Y, Muramatsu D, Echigo T, Yagi Y. GEINet: View-invariant gait recognition using a convolutional neural network. 2016 International Conference on Biometrics (ICB). IEEE; 2016. pp. 1–8.

[10] Yoo J-H, Hwang D, Moon K-Y, Nixon MS. Automated Human Recognition by Gait using Neural Network. 2008 First Workshops on Image Processing Theory, Tools and Applications. IEEE; 2008. pp. 1–6.

[11] Phinyomark A, Petri G, Ibáñez-Marcelo E, Osis ST, Ferber R. Analysis of big data in gait biomechanics: current trends and future directions. J Med Biol Eng. 2018;38: 244–260. 10.1007/s40846-017-0297-2 29670502 PMC5897457

[12] Chau T. A review of analytical techniques for gait data. Part 2: neural network and wavelet methods. Gait Posture. 2001;13: 102–120. 10.1016/s0966-6362(00)00095-3 11240358

[13] Halilaj E, Rajagopal A, Fiterau M, Hicks JL, Hastie TJ, Delp SL. Machine learning in human movement biomechanics: Best practices, common pitfalls, and new opportunities. J Biomech. 2018;81: 1–11. 10.1016/j.jbiomech.2018.09.009 30279002 PMC6879187

[14] Liu S, Wang X, Liu M, Zhu J. Towards better analysis of machine learning models: A visual analytics perspective. Visual Informatics. 2017;1: 48–56. 10.1016/j.visinf.2017.01.006

[15] Bach S, Binder A, Montavon G, Klauschen F, Müller K-R, Samek W. On Pixel-Wise Explanations for Non-Linear Classifier Decisions by Layer-Wise Relevance Propagation. PLoS One. 2015;10: e0130140. 10.1371/journal.pone.0130140 26161953 PMC4498753

[16] Montavon G, Lapuschkin S, Binder A, Samek W, Müller K-R. Explaining nonlinear classification decisions with deep Taylor decomposition. Pattern Recognit. 2017;65: 211–222. 10.1016/j.patcog.2016.11.008

[17] Arras L, Horn F, Montavon G, Müller K-R, Samek W. What is relevant in a text document?": An interpretable machine learning approach. PLoS One. 2017;12: e0181142. 10.1371/journal.pone.0181142 28800619 PMC5553725

[18] Baltich J, Emery CA, Stefanyshyn D, Nigg BM. The effects of isolated ankle strengthening and functional balance training on strength, running mechanics, postural control and injury prevention in novice runners: design of a randomized controlled trial. BMC Musculoskelet Disord. 2014;15: 407. 10.1186/1471-2474-15-407 25471989 PMC4295291

[19] Söderkvist I, Wedin PA. Determining the movements of the skeleton using well-configured markers. J Biomech. 1993;26: 1473–1477. 10.1016/0021-9290(93)90098-y 8308052

[20] von Tscharner V. Intensity analysis in time-frequency space of surface myoelectric signals by wavelets of specified resolution. J Electromyogr Kinesiol. 2000;10: 433–445. 10.1016/s1050-6411(00)00030-4 11102846

[21] Lapuschkin S, Binder A, Montavon G, Müller KR. The LRP toolbox for artificial neural networks. The Journal of Machine Learning Research. 2016;17: 3938–3942.

[22] Horst F, Mildner M, Schöllhorn WI. One-year persistence of individual gait patterns identified in a follow-up study—A call for individualised diagnose and therapy. Gait Posture. 2017;58: 476–480. 10.1016/j.gaitpost.2017.09.003 28926814

[23] Schöllhorn WI, Nigg BM, Stefanyshyn DJ, Liu W. Identification of individual walking patterns using time discrete and time continuous data sets. Gait Posture. 2002;15: 180–186. 10.1016/s0966-6362(01)00193-x 11869912

[24] Janssen D, Schöllhorn WI, Lubienetzki J, Fölling K, Kokenge H, Davids K. Recognition of emotions in gait patterns by means of artificial neural nets. J Nonverbal Behav. 2008;32: 79–92. 10.1007/s10919-007-0045-3

[25] Eskofier BM, Federolf P, Kugler PF, Nigg BM. Marker-based classification of young-elderly gait pattern differences via direct PCA feature extraction and SVMs. Comput Methods Biomech Biomed Engin. 2013;16: 435–442. 10.1080/10255842.2011.624515 22149087

[26] Hoerzer S, von Tscharner V, Jacob C, Nigg BM. Defining functional groups based on running kinematics using Self-Organizing Maps and Support Vector Machines. J Biomech. 2015;48: 2072–2079. 10.1016/j.jbiomech.2015.03.017 25869722

[27] Nigg BM, Vienneau J, Smith AC, Trudeau MB, Mohr M, Nigg SR. The preferred movement path paradigm: influence of running shoes on joint movement. Med Sci Sports Exerc. 2017;49: 1641–1648. 10.1249/MSS.0000000000001260 28277405

[28] Nigg BM, Mohr MM, Nigg SR. Muscle tuning and preferred movement path–a paradigm shift. CISS. 2017;2: 1–12.

[29] Horst F, Kramer F, Schäfer B, Eekhoff A, Hegen P, Nigg BM, et al. Daily changes of individual gait patterns identified by means of support vector machines. Gait Posture. 2016;49: 309–314. 10.1016/j.gaitpost.2016.07.073 27479216

[30] Baltich J, Emery C, Stefanyshyn D, Nigg B. The influence of ankle strength exercise training on running injury risk factors. Footwear Sci. 2015;7: S99–S100. 10.1080/19424280.2015.1038630

[31] Baltich J. The Effects of Resistance Strength Training and Functional Strength Training on Risk Factors for Running Injury. University of Calgary. 2016; 10.11575/prism/28115

[32] Baltich J, Emery CA, Whittaker JL, Nigg BM. Running injuries in novice runners enrolled in different training interventions: a pilot randomized controlled trial. Scand J Med Sci Sports. 2017;27: 1372–1383. 10.1111/sms.12743 27486011

[33] Almeida MO, Davis IS, Lopes AD. Biomechanical Differences of Foot-Strike Patterns During Running: A Systematic Review With Meta-analysis. J Orthop Sports Phys Ther. 2015;45: 738–755. 10.2519/jospt.2015.6019 26304644

[34] Giakas G, Baltzopoulos V. Time and frequency domain analysis of ground reaction forces during walking: an investigation of variability and symmetry. Gait Posture. 1997;5: 189–197. 10.1016/S0966-6362(96)01083-1

