# Supplementary material for: Individuality decoded by running patterns: Movement characteristics that determine the uniqueness of human running
Source: PLoS One. 2021 Apr 1;16(4):e0249657. doi: 10.1371/journal.pone.0249657 (PMC8016321; doi:10.1371/journal.pone.0249657)
Supplement: S1 File — This archive contains all the underlying data (raw and processed) presented in this publication and the respective analysis codes (Matlab) to follow the methodological steps of this work. (ZIP) [file pone.0249657.s001.zip › Data and analysis codes/Readme.docx]

This archive contains the underlying data and analysis codes for the submitted manuscript. The following files are included in this archive:

- Data.mat
- LRPanalysis.m
- Fig1.m
- Fig2.m
- Fig3and4.m
- Gliding_filter.m
- Lrp_toolbox-master

**Data.mat**

This file is a matlab data file containing the underlying data. The following variables are included:

- **isCorrect**: Array that indicates if a step pattern was correctly classified by the neural network. It is of dimension 931x1 because there were 931 step patterns collected on the second day of testing. A 1 indicates a correct classification while a 0 indicates a wrong classification.
- **Predictions**: Matrix representing the output of the neural network for each step pattern of the second day. Consequently, the dimensions of the matrix are 931x50. There were 931 step patterns classified and there were 50 participants each could have belonged to.
- **RAW**: Structure containing the raw data used for this study. The following fields exist within this structure:
  - **M:** Matrix with the raw data. Rows represent single step patterns and columns represent concatenated movement trajectories. There are 100 values per movement trajectories:
    - 1-100: Hip coronal
    - 101-200: Hip sagittal
    - 201-300: Hip transverse
    - 301-400: Knee coronal
    - 401-500: Knee sagittal
    - 501-600: Knee transverse
    - 601-700: Ankle coronal
    - 701-800: Ankle sagittal
    - 801-900: Ankle transverse
    - 901-1000: GRF medial / lateral
    - 1001-1100: GRF anterior / posterior
    - 1001-1200: GRF vertical
  - **day:** Array representing the day of data collection. 1 meaning day 1 and 2 meaning day 2. This array matches the rows of M and can be used to extract only step patterns of day one or day 2.
  - **grp:** Array representing the intervention groups. This array can be used to extract step patterns of specific intervention groups. 1: Balance group; 2: Strength group; 3: Control group.
  - **sbj:** Array representing the raw participant numbers. Note, these numbers are not linearly increasing, and they do range from 1 to 105. However, there are only 50 unique participants. In Matlab, use unique(sbj) to obtain the unique 50 participant numbers.
- **Relevance:** Matrix containing the unprocessed relevance scores obtained using Layer-wise relevance propagation. There is one relevance pattern for each step classified by the neural network. Rows represent steps, while columns follow the same structure as the matrix M.
- **Testing:** Structure containing the preprocessed inputs for the neural network that can be used for testing the model. X represents the input values and Y represents the target values.
- **Training:** Structure containing the preprocessed inputs for the neural network that can be used for training the model. X represents the input values and Y represents the target values.
- **nn:** The trained neural network structure.

**Lrp_toolbox-master**

This folder is contains the layer-wise relevance propagation codes for matlab from Lapuschkin S, Binder A, Montavon G, Müller KR. The LRP toolbox for artificial neural networks. The Journal of Machine Learning Research. 2016;17(1):3938–3942.

**Fig1.m**

The matlab script that creates the Figure 1 presented in the manuscript.

**Fig2.m**

The matlab script that creates the Figure 2 presented in the manuscript.

**Fig3and4.m**

The matlab script that creates the Figures 3 and 4 presented in the manuscript.

**LRPanalysis.m**

The matlab script that calculates the Relevance scores based on the raw data.

**Gliding_filter.m**

The lab-internal matlab function that applies the used smoothing process.
